# Determination of *n*-alkanes in *C. annuum* (bell pepper) fruit and seed using GC-MS: comparison of extraction methods and application to samples of different geographical origin

**DOI:** 10.1007/s00216-015-8755-6

**Published:** 2015-05-28

**Authors:** E. de Rijke, C. Fellner, J. Westerveld, M. Lopatka, C. Cerli, K. Kalbitz, C. G. de Koster

**Affiliations:** Mass Spectrometry of Biomacromolecules, Swammerdam Institute of Life Sciences (SILS), University of Amsterdam, Sciencepark 904, 1090 GE Amsterdam, The Netherlands; Earth Surface Science, Institute for Biodiversity and Ecosystem Dynamics, University of Amsterdam, Sciencepark 904, 1090 GE Amsterdam, The Netherlands; Korteweg-de Vries Institute, University of Amsterdam, Sciencepark 904, 1090 GE Amsterdam, The Netherlands; Netherlands Forensic Institute, P.O. Box 24044, 2490 AA The Hague, The Netherlands; Institute of Soil Science and Site Ecology, Technische Universität Dresden, Pienner Straße 19, 01737 Tharandt, Germany

**Keywords:** Extraction, GC-MS, *n*-Alkanes, Bell pepper, Seed, Geographical origin classification

## Abstract

An efficient extraction and analysis method was developed for the isolation and quantification of *n*-alkanes from bell peppers of different geographical locations. Five extraction techniques, i.e., accelerated solvent extraction (ASE), ball mill extraction, ultrasonication, rinsing, and shaking, were quantitatively compared using gas chromatography coupled to mass spectrometry (GC-MS). Rinsing of the surface wax layer of freeze-dried bell peppers with chloroform proved to be a relatively quick and easy method to efficiently extract the main *n*-alkanes C_27_, C_29_, C_31_, and C_33_. A combined cleanup and fractionation approach on Teflon-coated silica SPE columns resulted in clean chromatograms and gave reproducible results (recoveries 90–95 %). The GC-MS method was reproducible (*R*^2^ = 0.994–0.997, peak area standard deviation = 2–5 %) and sensitive (LODs, *S*/*N* = 3, 0.05–0.15 ng/μL). The total main *n*-alkane concentrations were in the range of 5–50 μg/g dry weight. Seed extractions resulted in much lower total amounts of extracted *n*-alkanes compared to flesh and surface extractions, demonstrating the need for further improvement of pre-concentration and cleanup. The method was applied to 131 pepper samples from four different countries, and by using the relative *n*-alkane concentration ratios, Dutch peppers could be discriminated from those of the other countries, with the exception of peppers from the same cultivar.

**Graphical Abstract:**
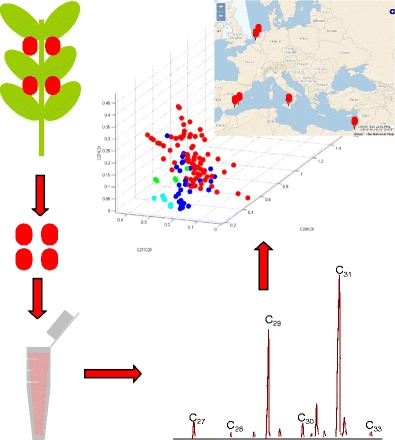
Procedure for pepper origin determination

Procedure for pepper origin determination

## Introduction

The Netherlands, after the USA, is the largest exporting country of agricultural products in the world [1]. Tomatoes, cucumbers, and peppers (*Capsicum annuum*) are the most important Dutch agricultural products. All aboveground parts of fruit and vegetable plants are covered with a hydrophobic cuticle, composed of cutin, a polyester of cross-linked hydroxy fatty acids in which surface waxes are embedded [2]. The fruit water loss and associated post-harvest quality of fruits and vegetables are dependent on the thickness of the epicuticular wax layer [3]. This wax layer consists of a mixture of long-chain aliphatic hydrocarbons, containing alkanes, fatty acids, primary and secondary alcohols, diols, ketones, and aldehydes [4]. The composition varies not only from species to species but also between different parts of the same plant [5]. The chemical composition of tomato surface wax has been extensively studied [2, 6–8], and recently, the lipid compositions of 50 pepper (*Capsicum*) cultivars were investigated [9]. The *n*-alkanes were the most abundant compounds in the wax layer of the pepper fruit. This layer was extracted by rinsing with chloroform, and the *n*-alkane profiles showed that *n*-C_27_, *n*-C_29_, and *n*-C_31_ were most abundant; their sum was between 30 and 550 μg/dm^2^ [9]. This is in the order of 0.1–1.7 mg on the total surface of one pepper, assuming that the average surface of a pepper is approx. 3 dm^2^. The *n*-alkane composition and concentrations are not likely to change during washing, handling, or transport, due to their hydrophobic nature. Previous research has shown that molecular distributions of *n*-alkanes can be used to determine the geographical origin of plant-based products, for example, for assessing the quality and authenticity of olive oils [10–12]. These studies indicated it was possible, using the *n*-alkane pattern and composition, to distinguish between crude and refined oils of different plants and geographical origins. By using principal component analysis and discriminant analysis, the specific patterns were shown to be significantly different, especially for crude oils, making it possible to use the *n*-alkane profiles as a means of determining the geographical origin and authenticity of the oils.

Due to their presence in the confined interior of the pepper fruit, pepper seeds are not likely to be influenced in chemical composition by the surroundings during growth. The general composition of pepper seed was described in several studies, reporting varying moisture content, e.g., as low as 6.25 % [13] and as high as 76.65 % [14]. In the latter study, the dry matter composition was reported as 19.28 % crude protein, 19.57 % crude fat, 4.88 % ash, and 56.28 % carbohydrates. In several other studies, pepper seed oil was investigated and found to consist mainly of triglycerides (∼90 %) and free fatty acids (5–10 %), while other compound groups, i.e., sterols, di/monoglycerides, triterpenes, phospholipids, and hydrocarbons, each constitute a minor part (∼1 %) [15–17]. In recent years, a number of studies were published on the free fatty acid composition of pepper seeds, with the main fatty acids being linoleic acid, oleic acid, palmitic acid, and stearic acid [15, 18, 19]. There are, however, limited studies on the chemical composition of the other compound groups for pepper seed, including the alkanes. In a recent paper, the alkane composition in pepper seeds is reported [20], but their study is limited to the analysis of the headspace fraction, sampled via solid-phase micro extraction. The main *n*-alkanes reported were eicosane (*n*-C_20_), tetradecane (*n*-C_14_), and nonadecane (*n*-C_19_), but no quantitative data was provided.

In the past decades, a large number of sample preparation techniques have been introduced especially suited for gas chromatography coupled to mass spectrometric (GC-MS) analysis, such as pressurized liquid extraction, commonly known as accelerated solvent extraction (ASE), and ultrasound extraction [21]. In our study, we compared these techniques, as well as ball mill extraction, shaking, and rinsing, for the extraction of *n*-alkanes from different parts of bell peppers. By means of ASE, samples are extracted under an elevated temperature, while an elevated pressure ensures that volatile extractants remain liquid [22]. Compared to classical Soxhlet extraction, with ASE, relatively small extraction volumes (typically 5–30 mL) are used, cycle times are less than an hour, and the system is automated. As an efficient extraction technique for lipids from organic material in soil and peat, ASE was investigated and compared to classical Soxhlet extraction [23, 24] and was applied to extract alkanes from barley leaf wax [25]. With ball mill extraction, an extraction vessel containing the extractant (dried, powdered) sample and stainless steel balls is placed on a rotor/stator element and, due to the high rotation speed of the rotor, the sample is drawn into the dispersion head and forced through the rotor/stator arrangement. The high accelerations of the sample and balls produce strong shear and thrust forces and high turbulence in the shear gap between rotor and stator, and provide mixing of the suspension. In this study, the above-mentioned extraction techniques, i.e., rinsing, ASE, ball mill extraction, ultrasonication, and shaking, were quantitatively compared for bell pepper fruit and seeds, using gas chromatography coupled to mass spectrometry (GC-MS). The optimized method was applied to quantify and compare the *n*-alkane profiles in bell peppers from important pepper-producing countries: the Netherlands, Spain, Italy, and Israel.

## Material and methods

### Pepper and seed samples

Dutch peppers were collected from two greenhouses in the Netherlands (Naktuinbouw, Roelofarendsveen; Helderman, Middenmeer). The peppers from Roelofarendsveen were from two different red bell pepper cultivars denoted NL RV 700 and NL RV 718. Spanish peppers were from greenhouses in Almeria (Enza Zaden), cultivar Tamarin, and Murcia (Instituto Murciano de Investigación y Desarrollo Agrario y Alimentario, IMIDA), cultivar Loreto. Italian (Sicily) peppers were bought in a local Italian Bio market, and Israeli peppers were from a Dutch supermarket (Albert Heijn, Amsterdam; Israeli origin was stated on the package); for both locations, the cultivar was unknown. For each greenhouse location, 20 peppers were sampled at different sampling dates throughout the season. Peppers from Roelofarendsveen were sampled each month from July 2013 to September 2014, and peppers from Middenmeer were sampled monthly from March to September 2014. Spanish peppers were sampled in May and August 2014 in both Murcia and Almeria; Israeli (six pieces) and Italian (five pieces) peppers were bought in May 2014. In total, 131 pepper samples were collected and analyzed from the four different countries.

### Extraction of pepper and seed samples

An overview of all pepper samples and extraction methods used in this study is presented in Table 1. The peppers were either used fresh as a whole or cut into pieces, collecting the seeds separately. The pepper pieces and seeds were freeze-dried in 3–5 days at a pressure below 50 mBar. The lyophilized pepper pieces were ground using a mortar and pestle and subsequently milled to a fine powder in a cutting mill (Retch 2M 200, Haan, Germany) at 10,000 rpm for 1 min. Lipid extraction was performed on freeze-dried powdered and fresh peppers and whole freeze-dried seeds. To each sample, internal standard (I.S., *n*-C_20_) was added prior to extraction. Each method was performed in duplicate, using two different peppers from one batch. And for each method, a blank sample (solvent plus I.S.) was included in the procedure.

**Table 1:** Overview of pepper/seed samples and extraction procedures

Sample type	Cultivar	Extraction technique	Solvent	FD/fresh	Amount of sample	Replicates	Pepper no	Results in the figures
Pepper	NL RV 700	Rinse	DCM/MeOH	Fresh	Whole pepper	1	P1	Fig. 1
		Rinse	Chloroform	Fresh	Whole pepper	1	P2	
	NL RV 718	Rinse	Chloroform	Fresh	¼ pepper	3	P1–P3	Figs. 2 and 5B
		ASE	DCM/MeOH	FD powder	1 g	3	P1–P2	
		ASE	Chloroform	FD powder	1 g	3	P1–P2	
	NL MM Tamarin	Shaking	Chloroform	FD powder	1 g	3	P1–P5	Fig. 4A
		Ultrasonication	Chloroform	FD powder	1 g	3	P1–P5	
		ASE	Chloroform	FD powder	1 g	3	P1–P5	
	SP AA Tamarin	Rinse	Chloroform	Fresh	½ pepper	3	P1–P3	Fig. 4B
		Rinse	Chloroform	FD whole	½ pepper	3	P1–P3	
	Various	Rinse	Chloroform	FD whole	Whole pepper	1	P1–P131	Fig. 6
Seed	NL RV 718	Ultrasonication	Chloroform	FD milled	3 g	2	P1, P3	Fig. 5B
		Ultrasonication	DCM/MeOH	FD milled	3 g	2	P1, P3	
	SP AA Tamarin	Ultrasonication	Chloroform	FD milled	1 g	3	P1–P3	Fig. 5A
		Shaking	Chloroform	FD milled	1 g	3	P1–P3	
		ASE	Chloroform	FD milled	1 g	3	P1–P3	
		Ball mill	Chloroform	FD milled	1 g	3	P1–P3	

*NL* Netherlands, *RV* Roelofarendsveen, *MM* Middenmeer, *SP* Spain, *AA* Almeria, *ASE* accelerated solvent extraction, *DCM* dichloromethane, *MeOH* methanol, *FD* freeze-dried

The two peppers of cultivar NL RV 700 were rinsed as a whole with DCM/MeOH (9:1, *v*/*v*) or chloroform. The three peppers of cultivar NL RV 718 were divided in four equal parts, and two quarters of each pepper were freeze-dried, milled, and extracted with ASE using either DCM/MeOH (93:7, *v*/*v*) or chloroform, while one fresh quarter of each pepper was extracted by rinsing with chloroform. The fresh seeds of two peppers from this batch were divided in two 3-g portions each and extracted in an ultrasonic water bath with either DCM/MeOH (9:1, *v*/*v*) or chloroform.

For the comparison of ASE, ultrasonication, and shaking with chloroform, 1-g samples were taken in triplicate from five Dutch peppers of one Middenmeer greenhouse batch (cultivar Tamarin) that were freeze-dried, milled, and combined. To compare chloroform rinse extraction of freeze-dried and fresh peppers, three Spanish peppers (Almeria, cultivar Tamarin) were cut in half and one half of the peppers was freeze-dried and the other half was used fresh. The combined freeze-dried and milled seeds of these peppers were used (1 g) to compare ultrasound extraction, extraction by shaking, ASE, and ball mill extraction.

Whole peppers were extracted by rinsing the skin of the fresh or freeze-dried pepper with 10 mL dichloromethane/methanol (DCM/MeOH, 9:1, *v*/*v*), chloroform, or hexane. The powder (1 g) was extracted using ASE, ultrasonication, or shaking. For ASE, an accelerated solvent extractor (ASE200, Thermo, Sunnyvale, USA) was used with a DCM/MeOH mixture (93:7, *v*/*v*) or chloroform at 100 °C and 103 bar (=1500 psi) for 20 min in three cycles (11 mL cells, 60 % flush volume), according to the procedure described in Jansen et al. [23, 24]. To check complete recovery, this procedure was repeated once on the extracted sample. It was decided to use two extraction cycles in the ASE protocol, as the second cycle still contained a minor part of the *n*-alkanes (∼5 %). For ultrasonication and shaking, 1 g of the powder was extracted in a vial with 10 mL chloroform in an ultrasonic bath for 10 min or on a shaking plate (Gerhardt Laboshake, Königswinter, Germany) for half an hour at 100 rpm. The extracts were filtered over a 0.45-μm PTFE (Teflon) syringe filter (Whatman, Dassel, Germany).

Fresh pepper seeds (3 g) were extracted in a 10-mL vial with DCM/MeOH (9:1, *v*/*v*) or chloroform in an ultrasonic bath for 10 min. In a separate experiment, different seed extraction methods were compared, i.e., ultrasound extraction, extraction by shaking, ASE, and ball mill extraction (ULTRA-TURRAX Tube Drive system, Dijkstra Vereenigde, Lelystad, the Netherlands). For extraction by ultrasound or shaking, 1 g of freeze-dried milled seeds was added to glass vials containing 10 mL chloroform and 10 ng/μL internal standard; the vials were closed and placed either on a shaking plate for half an hour at 100 rpm or in an ultrasonic bath for 10 min. ASE was performed using the chloroform method described above, and for the ball mill extraction, 1 g of freeze-dried seeds was added to a dedicated ball mill vessel, to which 15 stainless steel balls, 10 mL chloroform, and 10 ng/μL internal standard were added. The vessel was closed and the sample was extracted for 5 min on the ball mill system. After extraction, all seed extracts were decanted and filtered over a 0.45-μm syringe filter.

### Fractionation *n*-alkanes

The extracts were fractionated on either self-packed silica gel columns or Strata SI-1 SPE columns (Phenomenex, Torrance, CA, USA) into three fractions denoted F1 (hydrocarbons, esters), F2 (esters, ketones), and F3 (alcohols, fatty acids). A self-packed silica gel column was constructed by filling a glass syringe with a small piece of combusted glass wool and ∼1.5 g silica gel (Grace, Columbia, MD, USA), which was activated at 450 °C for 10 h, then deactivated with 5 % *w*/*w* ultrapure water, prior to use. The column was rinsed with 6 mL acetone, 6 mL dichloromethane, and 6 mL hexane and consequently dried for at least 4 h at 50 °C. After pre-conditioning the column with 6 mL hexane, it was ready for use. Strata SI-1 SPE columns were obtained from Phenomenex (Torrance, CA, USA) and pre-conditioned with 6 mL acetone, 6 mL dichloromethane, and 6 mL hexane before use. F1 was eluted with 5.5 mL hexane, evaporated to dryness under a gentle stream of nitrogen, and re-dissolved in 1 mL hexane. F2 was eluted with 7.5 mL hexane/dichloromethane (4:1, *v*/*v*) and evaporated to dryness under a gentle stream of nitrogen, re-dissolved in 1 mL hexane/dichloromethane, and stored at −20 °C. For collection of F3, the SPE column was eluted with 7.5 mL of DCM/MeOH (9:1, *v*/*v*), and the eluent was evaporated to dryness, re-dissolved in 1 mL DCM/MeOH, and stored at −20 °C.

Before analysis of F2 and F3, a derivatization step was performed. For this, the evaporated sample was re-dissolved in 1 mL DCM/2-propanol (2:1, *v*/*v*), of which 200 μL was transferred to a new vial and evaporated to dryness under a gentle stream of nitrogen. To the vial, 50 μL BSTFA/TMCS (*N*,*O*-bis(trimethylsilyl)trifluoroacetamide and trimethylsilyl chloride, 9:1, *v*:*v*) and 50 μL of *n*-hexane were added and the vial was closed tightly and heated for 1 h at 70 °C. After cooling, the sample was evaporated to dryness under a gentle stream of nitrogen and re-dissolved in 200 μL *n*-hexane for qualitative GC-MS analysis. Constituents of the hydrocarbon fraction F1 were identified and quantified using GC-MS by comparison to an external *n*-alkane standard mixture (*n*-C_21_ to *n*-C_40_) and corrected for the peak area of the internal standard. The recovery and the reproducibility of the method (*n* = 10) were tested using a standard mixture containing 4 *n*-alkanes plus internal standard in concentrations of 15 ng/μL (*n*-C_18_, *n*-C_24_, and *n*-C_30_) and 20 ng/μL I.S. (*n*-C_20_).

### Gas chromatography-mass spectrometry

GC-MS was performed using a ThermoQuest Trace GC 2000 connected to a Finnigan Trace MS quadrupole mass spectrometer (ThermoFisher Scientific, Waltham, MA, USA). GC conditions: 1.0 μL on-column injection at 5 μL/s, 2 m Siltek deactivated pre-column (i.d. 0.53 mm) connected to a 30 m Rtx-5Sil MS column (Restek, i.d. 0.25 mm; film thickness 0.1 μm), and carrier gas helium at a flow of 0.8 mL/min. Temperature program: 50 °C (2 min) to 80 °C at 40 °C/min (hold 2 min), to 130 °C at 20 °C/min, to 350 °C at 4 °C/min (hold 10 min). MS conditions: full scan (*m*/*z* 50–650), cycle time 0.65 s, electron ionization (70 eV). For the analysis of the *n*-alkanes in F1, the relative amounts of the most abundant *n*-alkanes and their relative intensities were compared to an *n*-alkane standard mixture (*n*-C_21_ to *n*-C_40_). For quantification, calibration plots of *n*-C_25_ to *n*-C_33_ were constructed by injecting a dilution series of the *n*-C_25_ to *n*-C_33_ mixture, in concentrations of 0–25 ng/μL (five data points in triplicate). The main *n*-alkanes were quantified in the 131 pepper samples from the four different countries, and their relative ratios were plotted in a 3D graph using Matlab v. 2010b (The Mathworks, Natick, MA).

## Results and discussion

### Gas chromatography-mass spectrometry

**Fig. 1:**
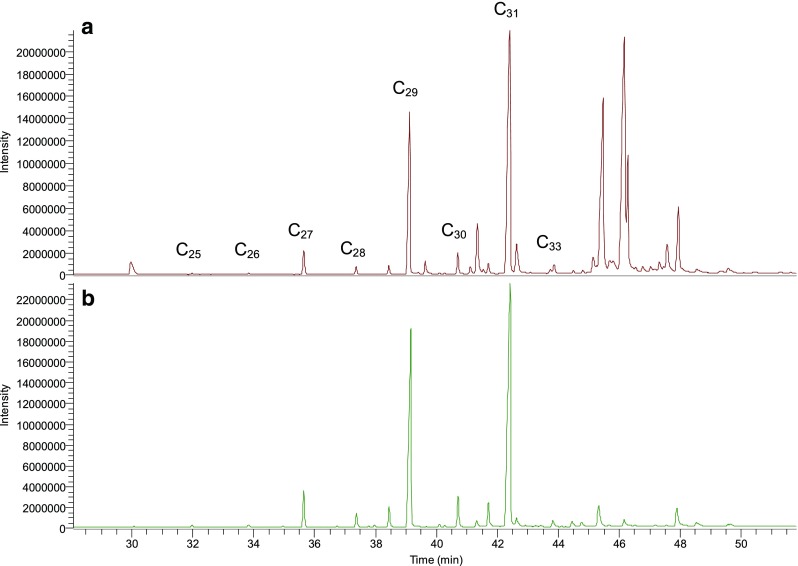
GC-MS TIC chromatograms of **A** F1 of DCM/MeOH rinse extract pepper 700-P1 and **B** F1 of chloroform rinse extract of pepper 700-P2

**Fig. 2:**
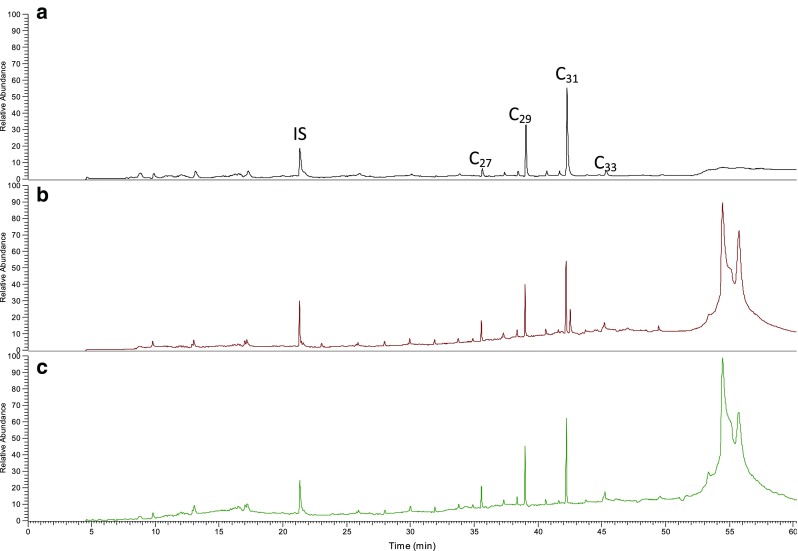
GC-MS TIC chromatograms of F1 of **A** chloroform rinse extract, **B** chloroform ASE extract, and **C** DCM/MeOH ASE extract of the same pepper

**Fig. 3:**
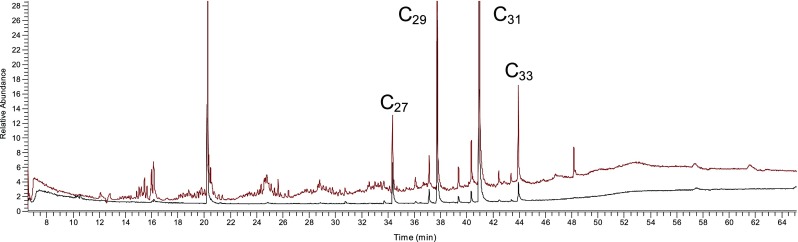
Chromatograms of F1 of a pepper rinse extract fractionated on a Strata SI-1 SPE normal cartridge (*red*) and a Teflon-coated cartridge (*black*)

The main constituents in the chloroform and DCM/MeOH rinse extracts (samples 700-P1 and P2, see Scheme 1), after cleanup on self-packed silica SPE columns, were identified as the *n*-alkanes C_27_, C_29_, C_31_, and C_33_, based on their mass spectrum and retention times compared to the C_21_–C_40_ reference standard. This result is in line with literature data [9, 26]. The relative intensities of the *n*-alkanes were very similar for both extracts (Fig. 1), but the DCM/MeOH extract contained a set of other compounds with retention times above 45 min, which were most likely terpenoids, of which the two main peaks were tentatively identified as amyrin and lupeol based on a NIST database search. In Fig. 2, the GC-MS chromatograms of F1 of three extracts of the same pepper (718-P1) are shown, using rinsing with either chloroform (Fig. 2A), ASE with chloroform (Fig. 2B), or ASE with DCM/MeOH (Fig. 2C). In order to simplify the protocol, fractionation of these samples was performed on Strata Si-1 SPE columns. Again, the relative intensities of the main *n*-alkanes were very similar using the three types of extraction, but all extracts showed an elevated baseline compared to the chromatograms of Fig. 1, which is more pronounced for the ASE extracts. The elevated baseline was most likely caused by the difference in cleanup procedure, i.e., using Strata Si-1 SPE columns instead of self-packed glass columns. It was hypothesized that the pre-conditioning step with acetone generates a background signal after Strata SPE cleanup, while for the self-packed columns, the acetone is removed during drying at 50 °C after conditioning of the columns. To test this hypothesis, the four-alkane standard mixture was applied in duplicate to two Strata SPE columns; one column was conditioned according to the protocol, and the other column was only conditioned with hexane. The latter resulted in a lower baseline and less background peaks between 6 and 17 min (data not presented). However, the samples fractionated on the Strata SPE columns only rinsed with hexane still showed a higher background signal, compared to the samples fractionated on the self-packed glass columns. Based on the polymeric nature of the peaks between 6 and 17 min and their corresponding mass spectra, it was assumed that this may be caused by the polypropylene material of the Strata Si-1 SPE columns. Therefore, an additional test was performed using the same Strata Si-1 SPE columns, of which the housing was coated with a Teflon layer. The extracts prepared with these Teflon SPE columns showed much cleaner chromatograms (Fig. 3). The recoveries of the standard mix using these Teflon-coated SPE columns were 90–95 %, with relative standard deviations of 8–10 %.

Analysis of F2 resulted in the tentative identification, based on a NIST library search, of various branched alkanes and alkenes and β-amyrin, α-amyrin, and lupeol. The compounds tentatively identified in F3 were the fatty acids and alcohols hexadecanoic acid, octadecan-1-ol, octadecanoic acid, β-amyrin, α-amyrin, lupeol, and friedelan-3-ol. These compounds were previously reported in literature [9, 26]. Fractions 2 and 3 did not contain any of the *n*-alkanes that were present in F1.

### Quantitative comparison between pepper extraction methods

*R*^2^ values of the GC-MS calibration plots were between 0.994 and 0.997, and limits of detection (*S*/*N* = 3) were between 0.05 and 0.15 ng/μL in all cases. The method was repeatable with relative standard deviations of the peak areas between 2 and 5 %.

**Fig. 4:**
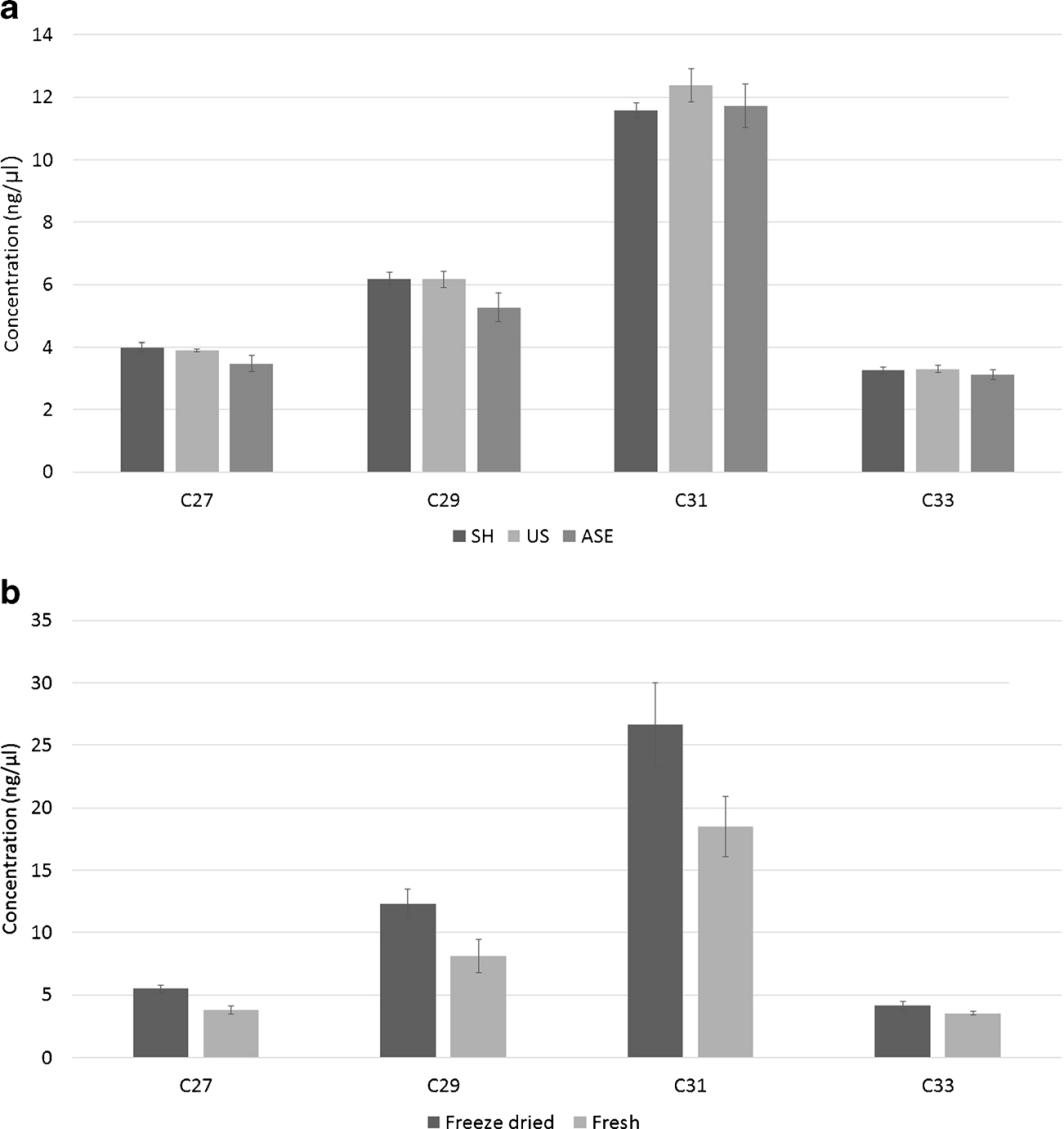
Comparison of chloroform-based extraction methods **A** accelerated solvent extraction (ASE), ultrasonication (US), and shaking (SH) of combined freeze-dried milled peppers P1–P5, NL MM Tamarin (*n* = 3), and **B** rinse extraction of freeze-dried and fresh peppers P1–P3, SP AA Tamarin (*n* = 3)

There is no significant difference between the recoveries and relative intensities of the main *n*-alkanes, *n*-C_27_, *n*-C_29_, *n*-C_31_, and *n*-C_33_ using extraction of freeze-dried milled peppers (718-P1, P2, P3 in Scheme 1) with shaking, ultrasonication, or ASE (Fig. 4A). With ASE, however, apart from *n*-alkanes, many other compounds were extracted from the samples with similar retention times, which means that an additional sample cleanup step would be needed for a selective *n*-alkane analysis.

Figure 4B shows the quantitative comparison between chloroform rinse extraction of freeze-dried and fresh peppers with *n*-C_20_ as an internal standard (three Spanish Tamarin peppers, Scheme 1). Rinsing freeze-dried peppers resulted in similar amounts and relative ratios of the extracted *n*-alkanes as rinsing fresh peppers. The advantage of freeze-dried samples is that they can be stored for a longer period at 4 °C without any degradation taking place. As most of the lipid compounds are in the wax on the surface of the pepper, rinsing is a good option to extract the *n*-alkanes from the skin. The estimated surface of an average-sized full-grown Dutch pepper (9 cm length and about 25 cm circumference) was about 3 dm^2^, containing approx. 25 μg/dm^2^*n*-C_31_, which is in the same order as reported in literature [9, 26, 27]. In our study, an approx. twofold difference in total *n*-alkane amounts of the four different cultivars was observed, which is in line with the results of Kissinger et al. [27] (1.7-fold difference between four cultivars from different, yet unknown, countries) and Bauer et al. [26] (twofold difference between 12 Dutch cultivars), while Parsons et al. [9] found an over tenfold difference in total *n*-alkane amounts between the *C. annuum* cultivars they investigated. The latter researchers, however, studied 31 worldwide cultivars.

Although several reviews have been published on the composition and biosynthesis of cuticular waxes of aerial parts of plants [28–30], published reports on cuticle composition of fruit, including bell pepper, are comparatively scarce [31], therefore limiting the comparison of our data to other studies.

### Quantitative comparison between seed extraction methods

**Fig. 5:**
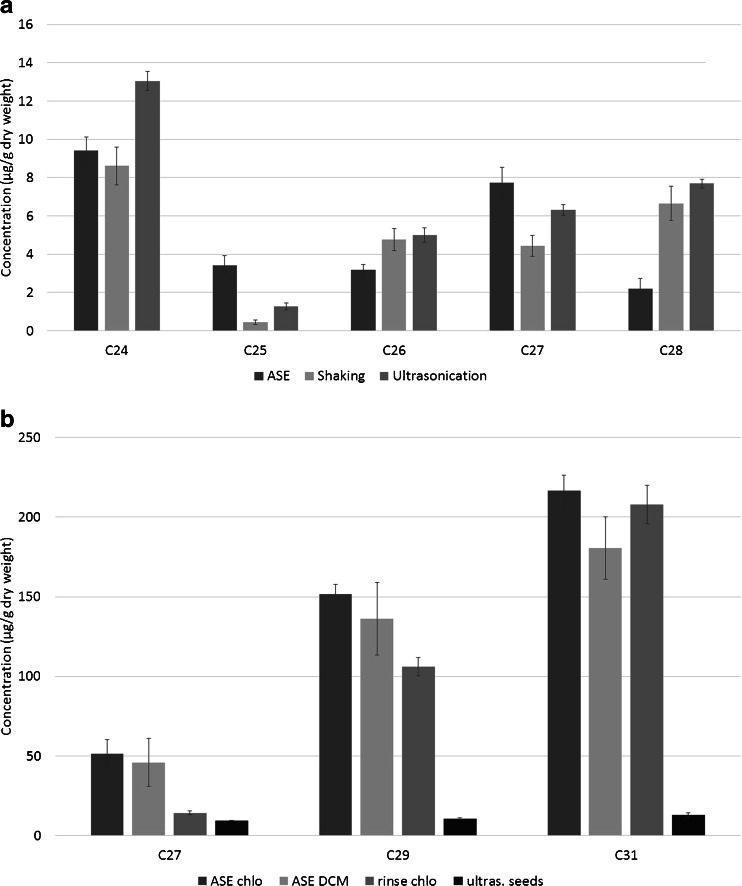
Quantitative comparison of extraction methods **A** ASE, shaking, and ultrasonication with chloroform of seeds from batch SP AA Tamarin (*n* = 3) and **B** ASE with chloroform (*chlo*), ASE with DCM/MeOH (*DCM*) and chloroform rinsing of peppers, and ultrasonication (*ultras.*) with chloroform of seeds from one batch NL RV 718 (*n* = 3)

The total amount of seeds harvested per pepper varied largely (0.5–6 g per pepper) between locations and also within batches. Extraction of fresh seeds with ultrasonication using either DCM/MeOH (9:1) or chloroform resulted in *n*-alkane concentrations of 9–13 μg/g for the chloroform extract, which showed a lower background in the GC-MS chromatograms. Using freeze-dried milled seed (average moisture content, 46 %), the main *n*-alkanes extracted with shaking, ASE, and ultrasound using chloroform as a solvent were *n*-C_24_, *n*-C_25_, *n*-C_26_, *n*-C_27_, and *n*-C_28_, with concentrations varying between 0.5 and 13 μg per gram dry seed (Fig. 5A). The concentrations of *n*-C_29_, *n*-C_30_, and *n*-C_31_ were below the limit of detection (0.5 μg/g, *S*/*N* = 3) for these samples. C_24_ was the main *n*-alkane for all three extraction methods, with average concentrations of 9, 9, and 13 ng/μL for shaking, ASE, and ultrasound extraction, respectively. The total amounts of *n*-alkanes extracted from the seeds were, however, much lower than in the flesh and skin extracts. In the ball mill seed extracts, the *n*-alkanes could not be detected because the chromatograms contained a large background signal, which was also present in the blank samples. This high background was most likely a result of co-extraction of the polypropylene material of the extraction vessel. The use of Teflon-coated extraction vials could resolve this problem, but unfortunately, these were not readily available.

### Quantitative comparison of pepper samples

In Fig. 5B, an overview is presented of the quantitative main *n*-alkane data of the flesh and seed of one pepper using different extraction methods. For the chloroform rinse extraction, on average, 1.8 times more material (as expressed in dry weight) was used in comparison to the other methods. The results show that there are no significant differences between the *n*-alkane concentrations as expressed per gram dry weight in the extracts obtained by ASE and rinsing. Ultrasound extraction of the seeds resulted in 5–15 times lower *n*-alkane concentrations as compared to the other methods. The total amount of the extracted main *n*-alkanes was in the range of 10–200 μg/g dry weight, depending on the extraction method.

**Fig. 6:**
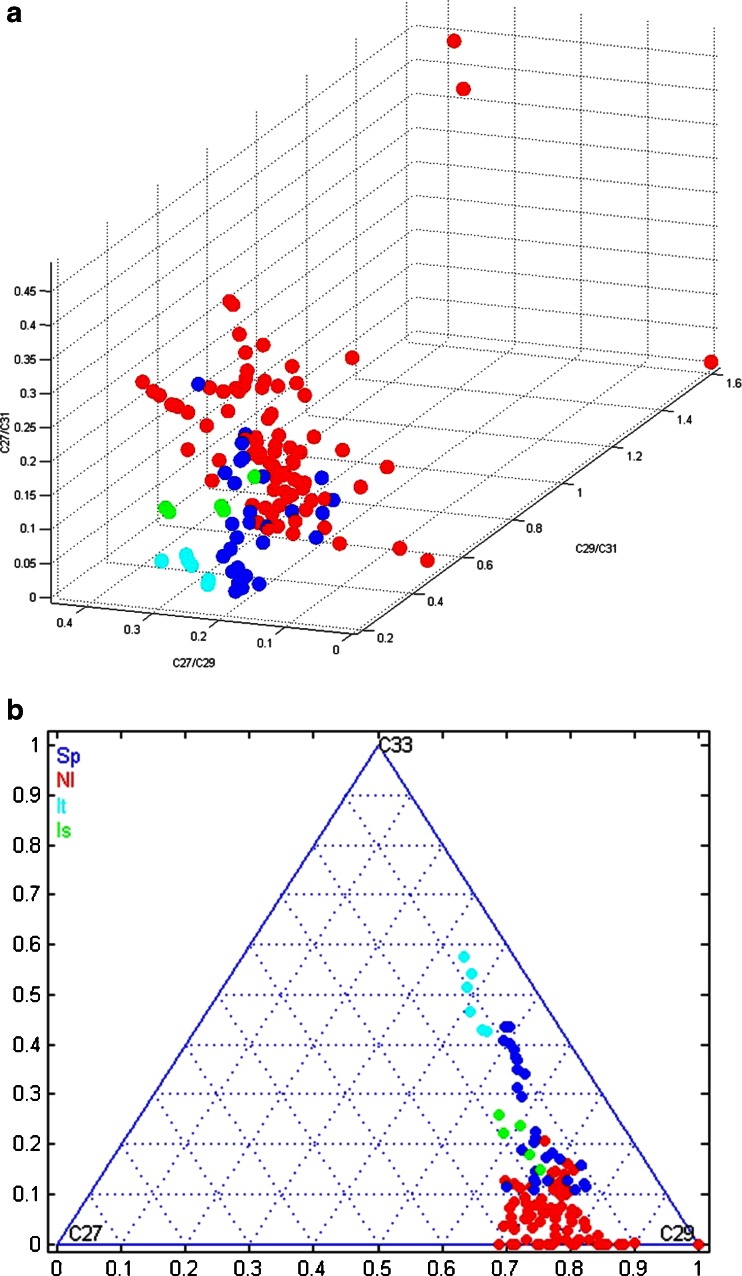
**A** 3D plot and **B** ternary plot of relative concentration ratios of alkanes in peppers from NL (*red*), SP (*blue*), IT (*light blue*), and IS (*green*)

The relative concentration ratios of the three main alkanes in the 131 pepper samples were plotted in a 3D graph as shown in Fig. 6A. As observed in the graph, pepper samples are clustering according to the country of origin. In some cases, however, Dutch and Spanish data points partly overlap. If the data are plotted in a ternary plot using Matlab as shown in Fig. 6B, a separation within the Spanish cluster is observed based on the two locations, Murcia and Almeria, of which the latter one partly overlaps with the Dutch peppers from Middenmeer. This can probably be explained by the fact that the Middenmeer and the Almerian peppers are from the same cultivar. The peppers from Murcia, Spain, were from the Loreto cultivar. To summarize, based on the relative concentration ratios of the three main *n*-alkanes, Dutch peppers could be discriminated from peppers from the other countries, with the exception of the peppers from Almeria.

## Conclusions

In this study, an efficient method for the determination of *n*-alkanes in bell pepper has been developed. The combination of freeze-drying the whole pepper, extraction by rinsing with chloroform, and a combined cleanup and fractionation approach on Teflon-coated silica SPE columns proved to be the optimal sample preparation method for these samples. The GC-MS method developed for quantification of the main *n*-alkanes was reproducible (*R*^2^ = 0.994–0.997, RSD peak area = 2–5 %) and sensitive (LODs, *S*/*N* = 3, 0.05–0.15 ng/μL). The relative concentration ratios of the three main alkanes in the pepper extracts showed clustering according to the country of origin. Adding additional data, using, e.g., compound-specific isotope ratio analysis methods, which we are currently developing, could further improve the classification power of the method. Extraction of pepper seeds resulted in much lower total amounts and different composition of *n*-alkanes compared to the pepper extracts and a higher background signal which disturbs quantification; therefore, this method needs further improvement.
